# Glycemic traits and Alzheimer’s disease: a Mendelian randomization study

**DOI:** 10.18632/aging.103887

**Published:** 2020-11-16

**Authors:** Yuesong Pan, Weiqi Chen, Hongyi Yan, Mengxing Wang, Xianglong Xiang

**Affiliations:** 1 Department of Neurology, Beijing Tiantan Hospital, Capital Medical University, Beijing, China; 2 China National Clinical Research Center for Neurological Diseases, Beijing, China

**Keywords:** Alzheimer’s disease, glycemic traits, insulin resistance, Mendelian randomization, diabetes

## Abstract

Previous observational studies have reported an association between impaired glucose metabolism and Alzheimer’s disease. This study aimed to examine the causal association of glycemic traits with Alzheimer’s disease. We used a two-sample Mendelian randomization approach to evaluate the causal effect of six glycemic traits (type 2 diabetes, fasting glucose, fasting insulin, hemoglobin A1c, homeostasis model assessment- insulin resistance and HOMA-β-cell function) on Alzheimer’s disease. Summary data on the association of single nucleotide polymorphisms with these glycemic traits were obtained from genome-wide association studies of the DIAbetes Genetics Replication And Meta-analysis and Meta-Analyses of Glucose and Insulin-related traits Consortium. Summary data on the association of single nucleotide polymorphisms with Alzheimer’s disease were obtained from the International Genomics of Alzheimer's Project. The Mendelian randomization analysis showed that 1-standard deviation higher fasting glucose and lower HOMA-β-cell function (indicating pancreatic β-cell dysfunction) were causally associated with a substantial increase in risk of Alzheimer’s disease (odds ratio=1.33, 95% confidence interval: 1.04-1.68, p=0.02; odds ratio=1.92, 95% confidence interval: 1.15-3.21, p=0.01). However, no significant association was observed for other glycemic traits. This Mendelian randomization analysis provides evidence of causal associations between glycemic traits, especially high fasting glucose and pancreatic β-cell dysfunction, and high risk of Alzheimer's disease.

## INTRODUCTION

Alzheimer’s disease is a major and increasing global health challenge in elderly people without available curative treatment [1]. Although the pathophysiologic mechanism of Alzheimer’s disease is largely unknown, impaired glucose metabolism in the brain may contribute to Alzheimer’s diseases pathology [2]. Recently, several epidemiological studies have suggested that some modifiable risk factors, such as type 2 diabetes and insulin resistance, were associated with cognitive decline or Alzheimer’s disease [3–7]. However, positive association was not observed in another study [8]. Furthermore, observational studies might be confounded by potential confounders and reverse causation [9]. Whether the association between glucose metabolism and Alzheimer’s disease observed in observational studies reflect causal association needs further investigation. Therefore, the causal association between impaired glucose metabolism and Alzheimer’s disease is still controversial.

Mendelian randomization (MR), using genetic variants as instrumental variables, is a method that enables strong causal inference between a risk factor and a disease [9]. In this study, we aimed to use MR analysis to evaluate the causal association between glycemic traits and Alzheimer’s disease.

## RESULTS

**Figure 1:**
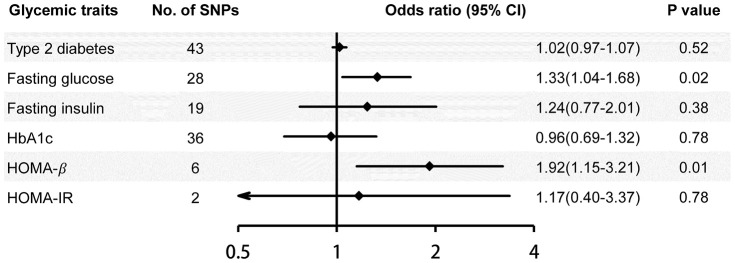
**Risk of Alzheimer’s disease for genetically predicted glycemic traits.** The associations are assessed using the inverse-variance weighted method. Estimates are per 1-unit higher log-odds of type 2 diabetes, 1-SD higher fasting glucose, fasting insulin and HOMA-IR, %-units higher HbA1c, and 1-SD lower HOMA-β (indicating pancreatic β-cell dysfunction). Trait values for fasting insulin, HOMA-β and HOMA-IR were naturally log transformed. CI, confidence interval; HbA1c, hemoglobin A1c; HOMA-β, homeostasis model assessment- β-cell function; HOMA-IR, homeostasis model assessment- insulin resistance.

**Figure 2:**
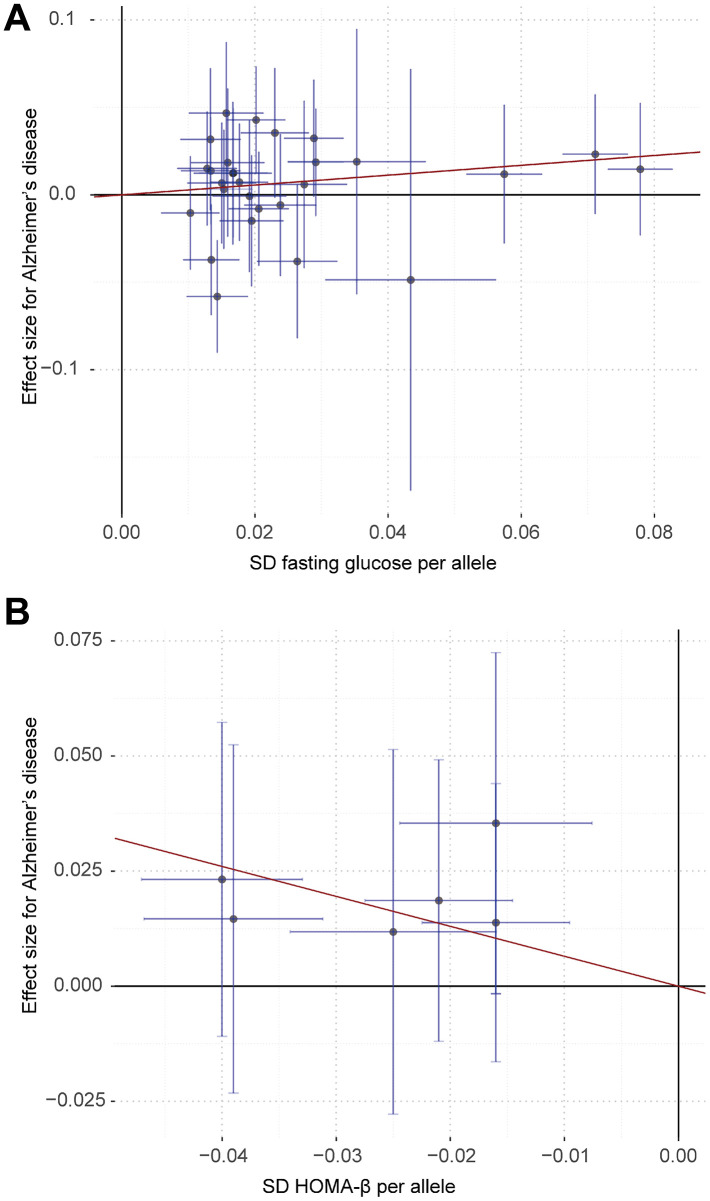
****Associations of fasting glucose (**A**) and HOMA-β (**B**) related variants with risk of Alzheimer’s disease. The red line indicates the estimate of effect using inverse-variance weighted method. Circles indicate marginal genetic associations between fasting glucose, HOMA-β and risk of Alzheimer’s disease for each variant. Error bars indicate 95% confidence intervals.

The inverse-variance weighted (IVW) method showed that 1-standard deviation (SD) higher fasting glucose and lower HOMA-β-cell function (HOMA-β) (indicating pancreatic β-cell dysfunction) were causally associated with a substantial increase in risk of Alzheimer’s disease (odds ratio (OR)=1.33, 95% confidence interval (CI): 1.04-1.68, p=0.02; OR=1.92, 95% CI: 1.15-3.21, p=0.01) (Figure 1). Similar association was observed in fasting glucose using the penalized robust IVW, weighted mode-based estimate (MBE) and Mendelian Randomization Pleiotropy RESidual Sum and Outlier (MR-PRESSO) methods, but not using the MR-Egger, simple median and weighted median methods of MR analyses (Table 1). For HOMA-β, similar results were observed using the penalized robust IVW, simple median and MR-PRESSO methods, but not using the MR-Egger, weighted median and weighted MBE methods of MR analyses. The results of leave-one-out sensitivity analyses showed that the associations between fasting glucose, HOMA-β and risk of Alzheimer’s disease were not substantially driven by any individual single nucleotide polymorphism (SNP). Association between each variant with fasting glucose, HOMA-β and risk of Alzheimer’s disease are displayed in Figure 2. No significant association of genetically predicted type 2 diabetes, fasting insulin, hemoglobin A1c (HbA1c) and homeostasis model assessment- insulin resistance (HOMA-IR) with risk of Alzheimer’s disease was observed using any MR method (Figure 1; Table 1).

**Table 1:** MR statistical sensitivity analyses.

			**Associations with AD**	
**Glycemic traits**	**MR methods**	**Parameter**	**OR/Odds (95% CI)**	**P value**
Type 2 diabetes	Penalized robust IVW	OR	1.01(0.96-1.07)	0.65
	MR-Egger	OR	0.98(0.84-1.14)	0.83
		Odds (intercept)	1.00(0.99-1.02)	0.63
	Simple median	OR	0.99(0.91-1.08)	0.83
	Weighted median	OR	0.99(0.91-1.07)	0.73
	Weighted MBE	OR	0.99(0.90-1.10)	0.89
	MR-PRESSO	OR	1.03(0.97-1.10)	0.35
Fasting glucose	Penalized robust IVW	OR	1.38(1.16-1.66)	<0.001
	MR-Egger	OR	1.36(0.78-2.38)	0.28
		Odds (intercept)	1.00(0.98-1.02)	0.91
	Simple median	OR	1.44(0.96-2.17)	0.08
	Weighted median	OR	1.24(0.90-1.70)	0.19
	Weighted MBE	OR	1.33(1.01-1.75)	0.04
	MR-PRESSO	OR	1.39(1.07-1.82)	0.02
Fasting insulin	Penalized robust IVW	OR	1.34(0.81-2.21)	0.25
	MR-Egger	OR	4.31(0.24-75.87)	0.32
		Odds (intercept)	0.98(0.94-1.03)	0.39
	Simple median	OR	1.35(0.69-2.62)	0.38
	Weighted median	OR	1.33(0.70-2.55)	0.39
	Weighted MBE	OR	1.49(0.61-3.62)	0.38
	MR-PRESSO	OR	1.24(0.83-1.87)	0.31
HbA1c	Penalized robust IVW	OR	0.96(0.70-1.32)	0.80
	MR-Egger	OR	1.21(0.55-2.64)	0.64
		Odds (intercept)	1.00(0.98-1.01)	0.51
	Simple median	OR	0.85(0.51-1.40)	0.52
	Weighted median	OR	1.00(0.61-1.64)	0.98
	Weighted MBE	OR	0.96(0.55-1.67)	0.87
	MR-PRESSO	OR	0.96(0.67-1.37)	0.81
HOMA-β	Penalized robust IVW	OR	1.85(1.24-2.77)	0.003
	MR-Egger	OR	0.91(0.22-3.79)	0.90
		Odds (intercept)	1.02(0.98-1.06)	0.27
	Simple median	OR	2.06(1.04-4.05)	0.04
	Weighted median	OR	1.73(0.93-3.22)	0.08
	Weighted MBE	OR	1.68(0.92-3.06)	0.09
	MR-PRESSO	OR	1.92(1.35-2.71)	0.01

MR, Mendelian randomization; AD, Alzheimer’s disease; OR, odds ratio; CI, confidence interval; IVW, inverse-variance weighted; MBE, mode-based estimate; MR-PRESSO, Mendelian Randomization Pleiotropy RESidual Sum and Outlier; HbA1c, hemoglobin A1c; HOMA-β, homeostasis model assessment -β-cell function. Estimates are per 1-unit higher log-odds of type 2 diabetes, 1-SD higher fasting glucose and fasting insulin, %-units higher HbA1c and 1-SD lower HOMA-β (indicating pancreatic β-cell dysfunction). Trait values for fasting insulin and HOMA-β were naturally log transformed. Sensitivity analyses were not performed for HOMA-IR since these methods requires >2 variants.

MR-Egger regression showed no evidence of directional pleiotropy for the association of any glycemic trait with Alzheimer’s disease (all p values for intercept >0.05) (Table 1). There was no evidence of heterogeneity in the IVW analysis for fasting glucose (Q=32.71, P=0.17), fasting insulin (Q=13.09, P=0.79), HbA1c (Q=45.20, P=0.12) and HOMA-β (Q=2.28, P=0.81), but heterogeneity was observed for type 2 diabetes (Q=67.91, P=0.007).

## DISCUSSION

Using two-sample MR analysis based on data from large-scale genome-wide association studies (GWAS), our study provided genetic evidence in supporting that glycemic disorder may lead to Alzheimer’s disease. In the present study, genetically predicted higher level of fasting glucose and lower level of HOMA-β (indicating pancreatic β-cell dysfunction) were causally associated with an increased risk of Alzheimer’s disease. However, no significant association was observed between type 2 diabetes, fasting insulin, HbA1c, HOMA-IR and risk of Alzheimer’s disease. Sensitivity analyses with different statistical models showed almost similar results.

Impaired glucose metabolism, which is modifiable, contributes to Alzheimer’s disease pathogenesis. It was even proposed that Alzheimer’s disease may be a brain-specific form of diabetes mellitus, a “type 3 diabetes” [10]. However, the precise mechanisms involved in the association of glucose metabolism with cognitive decline and Alzheimer's disease are not yet fully understood. Several assumed mechanisms have been proposed. Hyperglycemia may induce increased peripheral utilization of insulin and reduce insulin transport into the brain, ultimately producing brain insulin deficiency [11]. Impaired glucose metabolism, such as type 2 diabetes and insulin resistance, may lead to impaired neuronal insulin signaling, neuroinflammation, oxidative stress, resulting in amyloid-β accumulation, tau hyper-phosphorylation and subsequent cognitive decline and Alzheimer's disease [12, 13]. Recent studies showed that diabetes and insulin resistance were also associated with brain atrophy and aberrant functional connectivity [14, 15].

In the last decades, accumulating observational evidence has demonstrated that type 2 diabetes and insulin resistance were associated with cognitive decline or Alzheimer’s disease [3–7]. A meta-analysis showed that diabetes mellitus was associated with a 1.6-fold higher risk of Alzheimer’s disease [3]. In the English Longitudinal Study of Ageing study with 5189 participants and 10-year follow-up, HbA1c levels and diabetes status were associated with long-term cognitive decline [4]. Other longitudinal studies from Finland also observed significant association between insulin resistance and long-term cognitive decline [6, 7]. However, the association was nonsignificant between type 2 diabetes, insulin resistance or HbA1c and cognitive state or Alzheimer’s disease in a recent British cohort study and previous MR analyses [8, 16, 17]. The present study further emphasized the causal effect of high fasting glucose, pancreatic β-cell dysfunction on high risk of Alzheimer’s disease. This also indicated that the prediabetic stage could be a potential time-point for preventative treatment of Alzheimer’s disease. This result was supported by a recent study with 34year follow-up that found an U-shaped relationship between fasting insulin and dementia and excess risk for dementia in subjects with low level of insulin [18]. Potential explanation of the negative associations for fasting insulin and HOMA-IR in our study included weak instruments and small sample size. Furthermore, this might be caused by that HOMA-IR mainly reflect hepatic insulin resistance instead of brain insulin resistance [19].

The strength of the study is the design of MR analysis based on large-scale GWAS studies. The MR design uses genetic variants as instrumental variables to estimate the association between an exposure and a disease. As genetic variants are randomly allocated at meiosis and independent of other factors that may bias observational studies, MR analysis is not prone to potential reverse causation and unmeasured confounders, such as dietary and lifestyle preference, and thus can strengthen the evidence for causal inference [9]. Using two-sample MR method, we were able to test the effect of glycemic traits on Alzheimer's disease based on data from a large-scale cohort (17,008 cases and 37,154 controls). Nevertheless, potential bias may exist due to the nature of two-sample MR method since data of associations of SNP-exposure and SNP-outcome were derived from 2 different populations. Results in previous one-way or two-way MR analyses showed nonsignificant association between type 2 diabetes or fasting insulin with Alzheimer’s disease [16, 17, 20], but were not consistent for fasting glucose [16, 17, 21]. Another MR study found insulin sensitivity polygenic score formed from a subset of type 2 diabetes associated SNPs, but not the overall type 2 diabetes polygenic score, was causally associated with Alzheimer’s disease [22]. Our study distinguished from previous MR studies [16, 17, 20–22] by comprehensive evaluation of six glycemic traits and strict selection of instruments *via* exclusion of SNPs with potential pleiotropic effects.

Our study has several limitations. First, our analyses were performed in the population of European ancestry and may not be generalized to those of non-European ancestry. However, recent studies provided evidence of shared genetic architecture for glycemic diseases across ethnic groups [23]. The uniformity of ancestry minimizes the risk of bias by population admixture. Second, the estimates may still be biased by potential for residual pleiotropy (SNPs affect the outcome *via* a different pathway other than the exposure) [24]. However, we had a strict exclusion of instruments with potential pleiotropy and pleiotropic effect was not observed in MR-Egger regression. Third, further studies with large sample size are needed since the results were not validated in some sensitivity analyses in our study.

In conclusion, our two-sample MR analysis provides evidence of causal associations between glycemic traits, especially hyperglycemia and pancreatic β-cell dysfunction, and high risk of Alzheimer's disease. However, validating and replicating these findings in large-scale studies is warranted.

## MATERIALS AND METHODS

### Study design

**Figure 3:**
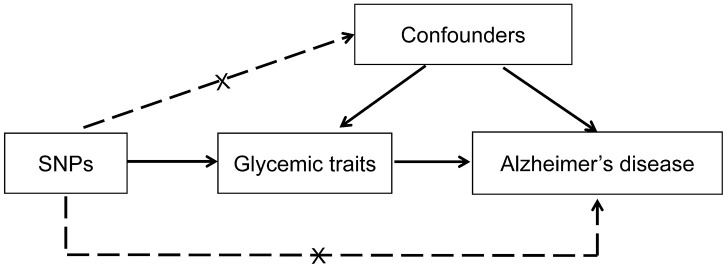
**Conceptual framework for the Mendelian randomization analysis of glycemic traits and risk of Alzheimer’s disease.** The design assumed that the genetic variants are associated with glycemic traits, but not with confounders, and the genetic variants are associated with risk of Alzheimer’s disease only through glycemic traits. SNP, single nucleotide polymorphism.

We designed a two-sample MR analysis to evaluate the causal effect of glycemic traits on Alzheimer’s disease (Figure 3). The MR design assumes that the genetic variants are associated with glycemic traits, but not with other confounders, and the genetic variants are conditionally independent of Alzheimer’s disease given glycemic traits and the confounding factors. Using the genetic variants as instrumental variables to assess the association between an exposure and an outcome, MR approach is a method that strengthens causal inferences and addresses susceptibility to potential confounding and reverse causation prone to conventional observational studies [9]. Six glycemic traits, including type 2 diabetes, fasting glucose, fasting insulin, HbA1c, HOMA-IR and HOMA-β, were evaluated. Data on the associations of SNPs with these glycemic traits and Alzheimer’s disease were obtained from recently published large-scale GWAS (Table 2) [25–30]. The protocols of the original studies were approved by the institutional review board of participating sites and informed consents were obtained from all participants.

**Table 2:** Characteristics of the GWAS used in this study.

**Phenotype**	**Consortium**	**Sample size**	**Ancestry**	**Genotype data**	**PMID**
Exposure (glycemic traits)					
Type 2 diabetes	DIAGRAM	Up to 212,747 individuals	European	GWAS array and metabochip array	22885922, 28566273
Fasting glucose	MAGIC	Up to 133,010 individuals	European	GWAS array and metabochip array	22885924
Fasting insulin	MAGIC	Up to 108,557 individuals	European	GWAS array and metabochip array	22885924
HbA1c	MAGIC	Up to 123,491 individuals	European	GWAS array	28898252
HOMA-β	MAGIC	Up to 98,372 individuals	European	GWAS array	20081858
HOMA-IR	MAGIC	Up to 94,636 individuals	European	GWAS array	20081858
Outcomes					
Alzheimer’s disease	IGAP	Up to 54,162 individuals	European	GWAS array	24162737

HbA1c, hemoglobin A1c; HOMA-β, homeostasis model assessment -β-cell function; HOMA-IR, homeostasis model assessment -insulin resistance.

### Instruments

Genetic variants which were associated with these six glycemic traits were obtained from previous published GWAS (Table 2). A total of 51 SNPs associated with type 2 diabetes were obtained from recently published two GWAS meta-analyses from the DIAbetes Genetics Replication And Meta-analysis (DIAGRAM) consortium [25, 26]. The first study combined the DIAGRAMv3 GWAS meta-analysis with a stage 2 meta-analysis of Metabochip, including 34,840 cases and 114,981 controls, overwhelmingly (97.6%) of European ancestry [25]. In that study, 38 genetic loci with at least 1 genetic variant associated with type 2 diabetes were identified (*P*< 5.0×10^−8^). The second study was an expanded GWAS of type 2 diabetes in Europeans, including a GWAS stage 1 with a total of 26,676 type 2 diabetes cases and 132,532 control participants from 18 GWAS and a Metabochip stage 2 with 14,545 type 2 diabetes cases and 38,994 controls from nonoverlapping 16 studies [26]. That study identified 13 novel type 2 diabetes -associated loci (*P*< 5.0×10^−8^).

SNPs associated with fasting glucose, fasting insulin, HbA1c, HOMA-IR and HOMA-β, were obtained from the recently published Meta-Analyses of Glucose and Insulin-related traits Consortium (MAGIC) [27–29]. MAGIC was a collaborative effort to combine data from multiple GWAS to identify loci that impact on glycemic and metabolic traits. In that GWAS meta-analysis, 36 genetic loci with at least 1 genetic variant associated with fasting glucose were identified in up to 133,010 non-diabetic participants of European ancestry from 66 studies, and 19 genetic loci with at least 1 genetic variant associated with fasting insulin in up to 108,557 individuals of European ancestry from 56 studies (*P*< 5.0×10^−8^) [27]. A total of 43 genetic loci with at least 1 genetic variant associated with HbA1c were identified in up to 123,665 non-diabetic individuals from 82 cohorts of European ancestry (*P*< 5.0×10^−8^) [28]. A total of 7 SNPs associated with HOMA-β and 2 SNPs associated with HOMA-IR were identified from GWAS meta-analysis of 46,186 non-diabetic participants from 21 studies with follow-up in up to 76,558 additional individuals of European ancestry (*P*< 5.0×10^−8^) [29]. Detailed information about the DIAGRAM and MAGIC consortia are showed in Supplementary Materials, Details of Studies and Participants.

All these SNPs were in different genomic regions and not in linkage disequilibrium with other SNPs in the same glycemic trait (r^2^<0.20). We performed a look-up of these SNPs in Phenoscanner to evaluate whether these SNPs were associated with other diseases or traits at genome-wide significance level (*P*< 5.0×10^−8^) which may indicate potential pleiotropic effects [31]. We found that eight SNPs for type 2 diabetes, eight SNPs for fasting glucose, six SNPs for HbA1c and one SNP for HOMA-β were also associated with other diseases or traits, such as white blood cell count, neutrophil count, low density lipoprotein cholesterol, high density lipoprotein cholesterol, triglycerides, total cholesterol, self-reported hypertension, coronary artery disease, years of educational attainment, serum urate (Supplementary Table 1). After exclusion of these SNPs and one SNP (rs12621844) for HbA1c not found in outcome datasets, we used the remaining 43 SNPs for type 2 diabetes, 28 SNPs for fasting glucose, 19 SNPs for fasting insulin, 36 SNPs for HbA1c, 6 SNPs for HOMA-β and 2 SNPs for HOMA-IR as the instrument in the MR analysis. Supplementary Table 2 shows the characteristics and associations of these included SNPs with the corresponding glycemic traits.

### Outcomes

Summary statistics for the associations between each glycemic trait- associated SNP and Alzheimer’s disease were obtained from the open-access GWAS of the International Genomics of Alzheimer's Project (IGAP) [30]. In brief, IGAP is a large two-stage GWAS on individuals of European ancestry [30]. In the first stage, IGAP mate-analyzed association of 7,055,881 SNPs with Alzheimer's disease in a total of 17,008 Alzheimer's disease cases and 37,154 controls of European ancestry from four GWAS datasets. In the second stage, IGAP genotyped and tested 11,632 SNPs for association with Alzheimer's disease in an independent set of 8,572 Alzheimer's disease cases and 11,312 controls. In that study, Alzheimer's Disease was defined as autopsy- or clinically-confirmed Alzheimer's Disease cases according to criteria, such as National Institute of Neurological and Communicative Disorders and Stroke and the Alzheimer's Disease and Related Disorders Association (NINCDS-ADRDA) criteria (Supplementary Materials, Details of Studies and Participants). We derived summarized data of the glycemic trait- associated SNPs from the dataset of stage 1 of the IGAP. The associations between each SNP related to glycemic traits and Alzheimer’s disease are presented in Supplementary Table 2.

### Statistical analysis

Two-sample MR approaches were used to compute estimates of each glycemic trait- Alzheimer’s disease association using summarized data of the SNP-glycemic trait and SNP-Alzheimer’s disease associations. The causal effect for each glycemic trait was evaluated by IVW method in which SNP-outcome coefficients were modeled as a function of SNP-exposure coefficients weighted by the inverse-variance of genetic associations with the outcome, assuming all SNPs were valid instruments [32]. In sensitivity analyses, we also performed complementary MR analyses using the penalized robust IVW, MR-Egger, simple median, weighted median, weighted MBE and MR-PRESSO methods for the traits of type 2 diabetes, fasting glucose, fasting insulin, HbA1c and HOMA-β. Sensitivity analyses were not performed for HOMA-IR since these methods require more than 2 variants. The penalized robust methods improved the robustness by penalizing the weights of candidate instruments with heterogeneous ratio estimates and providing robustness to outliers by performing robust regression [33]. The MR-Egger method was performed by the same weighted linear regression as IVW method but with the intercept unconstrained. The slope coefficient from the MR-Egger regression is a robust estimate of the causal effect against potential violations of assumptions due to directional pleiotropy (genetic variants affect the outcome *via* a different biological pathway from the exposure) [24]. The weighted median method used the weighted median ratio estimate with less weight given to outlying estimates and can provide a robust estimate even if up to 50% of instrumental variables are invalid [34]. The weighted MBE method used the mode of the IVW empirical density function as the effect estimate and can provide an estimate robust to horizontal pleiotropy [35]. The MR-PRESSO approach can detect and correct for pleiotropy *via* outlier removal in multi-instrument summary-level MR testing [36]. Heterogeneity between genetic variants was estimated by Q statistic in IVW method [34]. Potential pleiotropic effects were estimated by the values of intercept in MR-Egger regression. An intercept term that differs from zero indicates overall directional pleiotropy [24]. For the traits of fasting glucose and HOMA-β, we also performed a leave-one-out sensitivity analysis by leaving each genetic variant out of the MR analysis in turn to estimate the influence of outlying SNPs [37].

The associations between genetically predicted glycemic traits and Alzheimer’s disease were presented as ORs with their 95% CIs per 1-unit-higher log-odds of type 2 diabetes, 1-SD higher fasting glucose, fasting insulin and HOMA-IR, %-units higher HbA1c, and 1-SD lower HOMA-β (indicating pancreatic β-cell dysfunction), respectively. Trait values for fasting insulin, HOMA-IR and HOMA-β were naturally log transformed. The associations of each genetic variant with fasting glucose and HOMA-β were further plotted against their effects for the risk of Alzheimer’s disease. The threshold of statistical significance was 2-sided p-value <0.05. All analyses were performed using R version 3.5.3 (R Development Core Team). All data generated or analyzed during this study are included in this published article.

## Supplementary Material

Supplementary Materials 1

Supplementary Table 1

Supplementary Table 2
