# Supplementary material for: Glycemic traits and Alzheimer’s disease: a Mendelian randomization study
Source: Aging (Albany NY). 2020 Nov 16;12(22):22688–99. doi: 10.18632/aging.103887 (PMC7746331; doi:10.18632/aging.103887)
Supplement: Supplementary Materials 1 [file aging-12-103887-s001..pdf]

SUPPLEMENTARY MATERIAL 1

DETAILS OF STUDIES AND PARTICIPANTS

DIAbetes genetics replication and meta-analysis (DIAGRAM)

The DIAGRAM consortium is a grouping of researchers with shared interests in performing large-scale studies to characterize the genetic basis of type 2 diabetes (T2D), and a principal focus on samples of European descent. The membership and scope of DIAGRAM has developed as the scale of collaboration in the field has increased. The initial instance of DIAGRAM (retrospectively termed "DIAGRAM v1") enabled the combination of T2D genome wide association studies (GWAS) from the UK (WTCCC), DGI and FUSION groups. An incremental meta-analysis ("DIAGRAM v2" or "DIAGRAM+") added GWAS data from a further five studies (DGDG, KORA, Rotterdam, deCODE, EUROSPAN for a total of 8,130 cases and 38,987 controls) together with extensive replication involving 20 other cohorts. GWAS data from the Framingham, ARIC and NHS studies was only used for in silico replication, the full data from these studies was subsequently combined to constitute the largest current GWAS dataset in samples of European descent ("DIAGRAMv3": 12,171 cases and 56,862 controls). The present study combined the DIAGRAMv3 (stage 1) GWAS meta-analysis with a stage 2 meta-analysis comprising 22,669 cases and 58,119 controls genotyped with Metabochip, including 1,178 cases and 2,472 controls of Pakistani descent (PMID: 22885922).

An expanded GWAS of T2D in Europeans were performed with a GWAS stage 1 and Metabochip stage 2. The DIAGRAM stage 1 analyses comprised a total of 26,676 T2D cases and 132,532 control participants from 18 GWAS (ARIC, BioMe, deCODE, DGDG, DGI, EGCUT-370, EGCUT-OMNI, EPIC-InterAct, FHS, FUSION, GoDARTS, HPFS, KORAgen, NHS, PIVUS, RS-I, ULSAM, WTCCC). The Metabochip stage 2 follow up comprised 14,545 T2D case and 38,994 control subjects from 16 studies (D2D2007, DANISH, DIAGEN, DILGOM, DRsEXTRA, EMIL-Ulm, FUSION2, NHR, IMPROVE, InterACT-CMC, Leipzig, METSIM, HUNT/TROMSO, SCARFSHEEP, STR, Warren2/58BC) with Metabochip data, in which the participants did not overlap those included in stage 1. Stage 1 study sizes ranged between 80 and 7,249 T2D cases and from 455 to 83,049 controls. The Metabochip follow-up study sizes ranged from 101 and 3,553 T2D cases and from 586 to 6,603 controls. For SNVs not captured on Metabochip directly or by proxy, follow-up in 2,796 individuals with T2D and 4,601 controls from the EPIC-InterAct study was performed. In addition, 9,747 T2D cases and 61,857 controls from the GERA study were used to follow-up six low frequency variants not captured on Metabochip. All study participants were of European ancestry and were from the United States and Europe (PMID: 28566273).

Meta-analyses of glucose and insulin-related traits consortium (MAGIC)

MAGIC represents a collaborative effort to combine data from multiple GWAS to identify additional loci that impact on glycemic and metabolic traits. MAGIC investigators have initially studied fasting glucose, fasting insulin, 2h glucose and HbA1c, as well as performed meta-analysis of more sophisticated measures of insulin secretion and sensitivity.

GWAS meta-analysis data results for fasting glucose are from models adjusted for age and sex, and from up to 133,010 non-diabetic participants of European ancestry from 66 studies. Fasting insulin results are for ln-transformed fasting insulin as the outcome and are adjusted for age, sex and are reported both with and without BMI adjustment, and from up to 108,557 individuals of European ancestry from 56 studies (PUBMED: 22885924).

Ancestry-specific and transethnic genome-wide meta-analysis summary statistics for association with HbA1c in up to 159,940 individuals from 82 cohorts of European (N=123,665), African (N=7,564), East Asian (N=20,838) and South Asian (N=8,874) ancestry. All participants were free of diabetes. HbA1c trait values are untransformed and adjusted for age, sex and study-specific covariates (PMID: 28898252). Only data of European ancestry were used in the present analysis.

The fasting insulin and fasting glucose datasets were generated by performing a meta-analysis of up to 21 genome-wide association studies (GWAS) informative for fasting glucose, fasting insulin and indices of β-cell function (HOMA-β) and insulin resistance (HOMA-IR) in up to 46,186 non-diabetic participants. Follow-up of 25 lead SNPs were performed in up to 76,558 additional individuals of European ancestry. Fasting glucose trait values are not transformed. Trait values for fasting insulin, HOMA-IR, HOMA-β and fasting proinsulin have been naturally log transformed. All datasets are adjusted for age, sex and study-specific covariates (PMID: 20081858).

International Genomics of Alzheimer's Project (IGAP)

IGAP is a large two-stage study based upon GWAS on individuals of European ancestry. In stage 1, IGAP used

genotyped and imputed data on 7,055,881 SNPs to meta-analyse four GWAS datasets with a total of 17,008 Alzheimer's disease cases and 37,154 controls (The Alzheimer Disease Genetics Consortium [ADGC], The Cohorts for Heart and Aging Research in Genomic Epidemiology consortium [CHARGE], The European Alzheimer's disease Initiative [EADI], and The Genetic and Environmental Risk in AD consortium [GERAD]). In stage 2, 11,632 SNPs were genotyped and tested for association in an independent set of 8,572 Alzheimer's disease cases and 11,312 controls. The present study used the dataset of stage 1 of the IGAP (PMID: 24162737).

The investigators within IGAP contributed to the design and implementation of IGAP and/or provided data but did not participate in analysis or writing of this report. IGAP was made possible by the generous participation of the control subjects, the patients, and their families. The i–Select chips was funded by the French National Foundation on Alzheimer's disease and related disorders. EADI was supported by the LABEX (laboratory of excellence program investment for the future) DISTALZ grant, Inserm, Institut Pasteur de Lille, Université de Lille 2 and the Lille University Hospital. GERAD was supported by the Medical Research Council (Grant n° 503480), Alzheimer's Research UK (Grant n° 503176), the Wellcome Trust (Grant n° 082604/2/07/Z) and German Federal Ministry of Education and Research (BMBF): Competence Network Dementia (CND) grant n° 01GI0102, 01GI0711, 01GI0420. CHARGE was partly supported by the NIH/NIA grant R01 AG033193 and the NIA AG081220 and AGES contract N01–AG–12100, the NHLBI grant R01 HL105756, the Icelandic Heart Association, and the Erasmus Medical Center and Erasmus University. ADGC was supported by the NIH/NIA grants: U01 AG032984, U24 AG021886, U01 AG016976, and the Alzheimer's Association grant ADGC–10–196728.
