# Supplementary material for: Glycemic traits and Alzheimer’s disease: a Mendelian randomization study
Source: Aging (Albany NY). 2020 Nov 16;12(22):22688–99. doi: 10.18632/aging.103887 (PMC7746331; doi:10.18632/aging.103887)
Supplement: Supplementary Table 1 [file aging-12-103887-s002..pdf]

SUPPLEMENTARY TABLE

Supplementary Table 1. SNPs associated with other diseases or traits at genome-wide significance level ($P < 5.0 \times 10^{-8}$).

Glycemic traits	SNP	Associated other diseases or traits
T2D	rs10203174	Lymphocyte percentage of white cells, lymphocyte count
T2D	rs13389219	Waist hip ratio, triglycerides, body mass index
T2D	rs2943640	HDL cholesterol, triglycerides
T2D	rs7202877	Coronary artery disease
T2D	rs10401969	Total cholesterol, triglycerides, LDL cholesterol
T2D	rs9271774	White blood cell count, lymphocyte count, neutrophil count
T2D	rs635634	LDL cholesterol, total cholesterol, white blood cell count
T2D	rs2925979	HDL cholesterol, adiponectin, self-reported hypertension
FG	rs983309	Inflammation, total cholesterol, HDL cholesterol
FG	rs3829109	Neutrophil count, white blood cell count, granulocyte count
FG	rs9368222	Self-reported hypertension
FG	rs11715915	Years of educational attainment
FG	rs17762454	Serum urate, white blood cell count
FG	rs780094	Triglycerides, serum urate, white blood cell count, C reactive protein
FG	rs7944584	Self-reported hypertension
FG	rs174550	Triglycerides, LDL cholesterol
HbA1c	rs7616006	Lymphocyte count, monocyte count, total cholesterol, LDL cholesterol
HbA1c	rs9818758	Years of educational attainment
HbA1c	rs1800562	Self-reported hypertension
HbA1c	rs592423	Reticulocyte count, triglycerides, HDL cholesterol
HbA1c	rs579459	LDL cholesterol, total cholesterol, interleukin 6, coronary artery disease
HbA1c	rs10774625	Lymphocyte count, self-reported hypertension, coronary artery disease, total cholesterol
HOMA-β	rs174550	Triglycerides, LDL cholesterol, HDL cholesterol, white blood cell count

SNP, single nucleotide polymorphism; T2D, type 2 diabetes; FG, fasting glucose; HbA1c, hemoglobin A1c; HOMA-β, homeostasis model assessment -β-cell function; LDL, low density lipoprotein; HDL, high density lipoprotein.
